# Cholecystohepatic Duct: A Biliary Duct Variant Resulting in Postcholecystectomy Bile Leak—Case Report and Review of Normal and Common Variant Biliary Anatomy

**DOI:** 10.1155/2019/6812793

**Published:** 2019-06-17

**Authors:** Nathan Meyer, Sayf Al-Katib, Farnoosh Sokhandon

**Affiliations:** Beaumont Hospital, Oakland University William Beaumont School of Medicine, Department of Diagnostic Radiology and Molecular Imaging, 3601 W 13 Mile Rd, Royal Oak, MI 48073, USA

## Abstract

Although relatively infrequent, bile duct leaks are among the primary complications of hepatobiliary surgery and cholecystectomy given the large number of these operations performed annually around the world. Variant biliary anatomy increases the risk of surgical complications, especially if unrecognized on preoperative imaging or intraoperatively. Presented here is a case of a patient with an unrecognized cholecystohepatic duct at the time of surgery leading to bile leak after cholecystectomy. Numerous factors made for a technically difficult surgery with obscuration of the true anatomy, ultimately resulting in transection of the cholecystohepatic duct. Understanding normal and variant biliary anatomy will help prevent avoidable complications of hepatobiliary surgery.

## 1. Introduction

Bile duct leaks typically result from blunt abdominal trauma or iatrogenic injury. Despite being a rare complication of hepatobiliary surgery, their importance to radiologists and surgeons cannot be understated due to the frequency of these operations. With over 600,000 cases performed annually in the United States, this is especially true in the era of laparoscopic cholecystectomy which has increased the rate of bile duct injuries from 0.2% to 0.5% since becoming widely available [1]. Risk factors for bile duct injury during cholecystectomy include surgeon inexperience, variant anatomy, and sequelae of inflammatory changes around the gallbladder (GB) [1, 2].

## 2. Case Presentation

**Figure 1:**
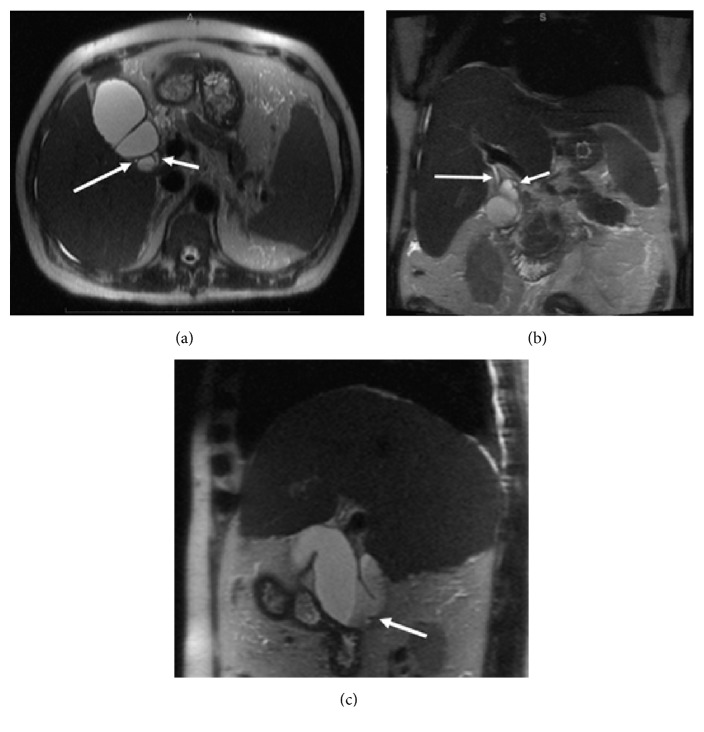
Preoperative thin slice MRCP images in axial (a), coronal (b), and sagittal planes (c). A normal cystic duct (CD) is seen at the GB neck medially (short arrows in (a), (b)). Arising from the posterior wall of the infundibulum is an accessory duct (long arrows in (a), (b)). Sagittal image demonstrates layering sludge within the dependent portions of the GB body and infundibulum as well as a hypointense gallstone (arrow in (c)).

A 53-year-old male presented to our institution's emergency room one week after returning from a trip abroad (Germany and India) with complaints of fever, fatigue, jaundice, shortness of breath, and back pain. He had a history of cholelithiasis and alcoholism. An ultrasound of the abdomen showed cholelithiasis/GB sludge without evidence of acute cholecystitis. Physical examination also elicited no tenderness on palpation of the abdomen. Laboratory work-up revealed elevated liver enzymes (alkaline phosphatase 497 U/L, aspartate transaminase 126 U/L, alanine transaminase 47 U/L) and elevated bilirubin (total 9.3 mg/dL and direct 5.8 mg/dL). There was no leukocytosis. The patient underwent magnetic resonance cholangiopancreatography (MRCP) examination which showed peripancreatic and pancreatic edema. This correlated with an elevated lipase to 591 U/L and he was diagnosed with acute interstitial edematous pancreatitis. It was also noted on MRCP that an accessory bile duct was present arising from the right hepatic ductal system and inserting into the infundibulum of the GB (Figure 1).

**Figure 2:**
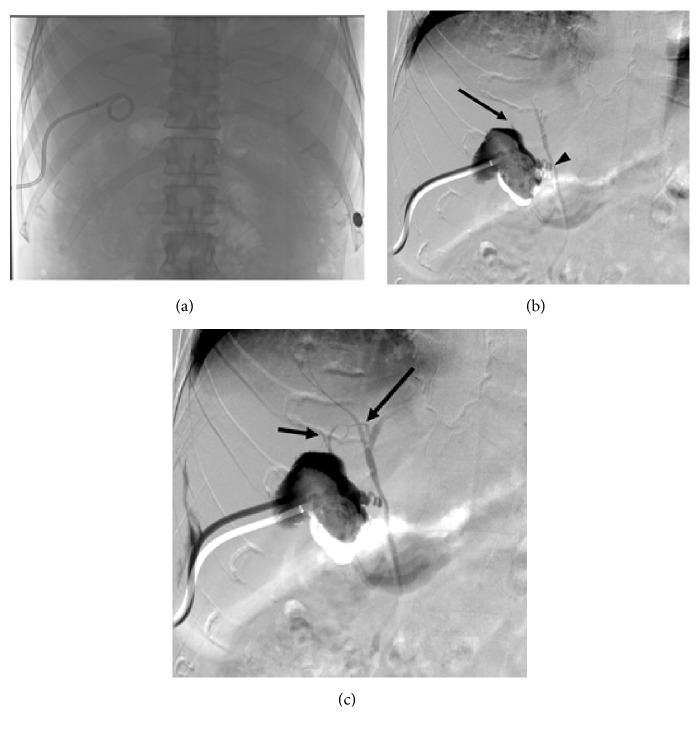
(a) Frontal fluoroscopic scout image of the PCT. (b, c) Digital subtraction cholecystography (cholecystogram). (b) Filling of the gallbladder via contrast injection through the PCT demonstrates early filling of the gallbladder as well as the common bile duct (CBD) and CHD via the CD (arrowhead). An additional duct is seen extending superiorly from the gallbladder (arrow). (c) The accessory duct eventually communicates with the right posterior duct (RPD) (long arrow). Contrast also refluxes into a smaller segmental duct extending from segment VII (short arrow) and feeds into the accessory cholecystohepatic duct.

Two days later the patient developed acute right upper quadrant (RUQ) pain and leukocytosis. He underwent hydroxy iminodiacetic acid (HIDA) scan which showed decreased hepatic uptake consistent with liver dysfunction, delayed biliary to bowel transit, and no filling of the gallbladder even on delayed images 24 hours after injection. These findings were concerning for obstruction of the cystic duct, thus acute cholecystitis. The patient was treated with antibiotics. He was not deemed a surgical candidate due to concomitant urosepsis, acute kidney injury, pancreatitis/hepatitis, and cholestatic jaundice with coagulopathy. A gastroenterologist was also following the patient; however ERCP was not performed due to lack of definitive evidence of cholangitis, biliary dilatation, or choledocholithiasis. A percutaneous cholecystostomy tube (PCT) was therefore placed by interventional radiology. During the PCT placement cholecystogram, the accessory bile duct was seen extending from the GB and connecting to the right posterior duct (RPD) (Figure 2). After a lengthy hospital admission, his condition stabilized and he was discharged home with the PCT in place.

Two months later, shortly after his PCT was removed, he again developed RUQ pain. CT scan redemonstrated gallstones within the GB neck. Cholecystectomy was indicated given the patient's clinical status. The surgery began under laparoscopic technique, but was converted to open surgery as there was poor visualization of the gallbladder fundus, rigidity of the liver secondary to cirrhosis, and a significant amount of adhesions. Despite description of the cholecystohepatic duct in the radiology reports, the same difficult factors resulted in its misidentification as the CD, leading to transection. Upon further dissection, another duct was seen entering the gallbladder, eventually being identified as the true CD on intraoperative cholangiogram. Both ducts were tied off with sutures, and no further leakage of bile was seen in the operating room. A Jackson Pratt drain was also left in the gallbladder fossa at the time of surgery. Prior to its discontinuation, only serosanguinous output was visualized, without evidence of bile leak.

**Figure 3:**
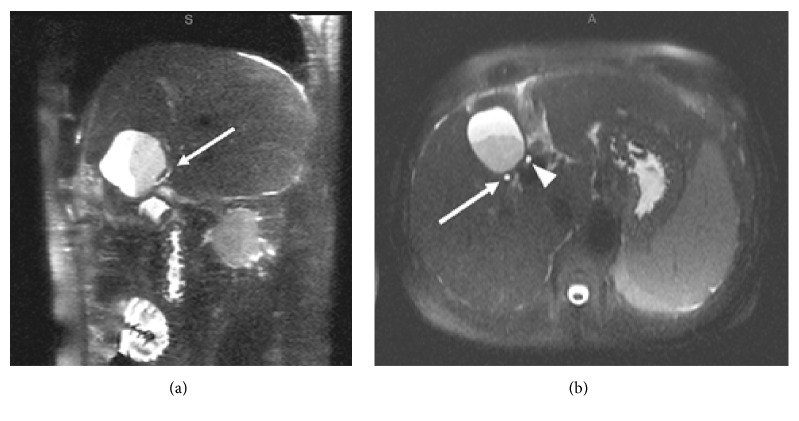
Postoperative thin slice MRCP images in sagittal (a) and axial (b) planes. Fluid collection with layering debris is present in the gallbladder fossa. (a) The cholecystohepatic duct terminates within the collection, entering posteriorly (arrow). (b) More superiorly, the duct is again seen posterior to the fluid collection (arrow). It is in a similar location compared to the preoperative imaging, but slightly more dilated. The common hepatic duct is also seen more medially (arrowhead).

During a follow-up US of the abdomen for liver disease approximately 6 weeks after surgery, a fluid collection with layering debris was seen in the gallbladder fossa. Subsequent MRCP (Figure 3) depicts the cholecystohepatic duct terminating in the fluid collection within the gallbladder fossa. The transection of this cholecystohepatic duct, despite being tied off with sutures, resulted in a bile leak likely due to its significant size (2-3 mm diameter) and partial continued drainage of the posterior right hepatic lobe. Given that this was an incidental finding and the patient was asymptomatic, conservative management was chosen. Serial follow-up ultrasounds showed decreasing size of the fluid collection and eventual spontaneous resolution.

## 3. Discussion

Although presurgical work-up of biliary disease typically includes imaging such as ultrasound, CT, and HIDA scans, these modalities offer limited anatomic detail of the intra and extrahepatic biliary ducts. On the contrary, modalities such as MRCP, endoscopic retrograde cholangiopancreatography (ERCP), percutaneous transhepatic cholangiogram, intraoperative cholangiogram, or cholecystogram during PCT placement offer excellent detail of the biliary anatomy and provide the opportunity to identify potential anatomical pitfalls for the surgeon. For this to be true, the radiologist must be familiar with problematic biliary anatomic variations.

**Figure 4:**
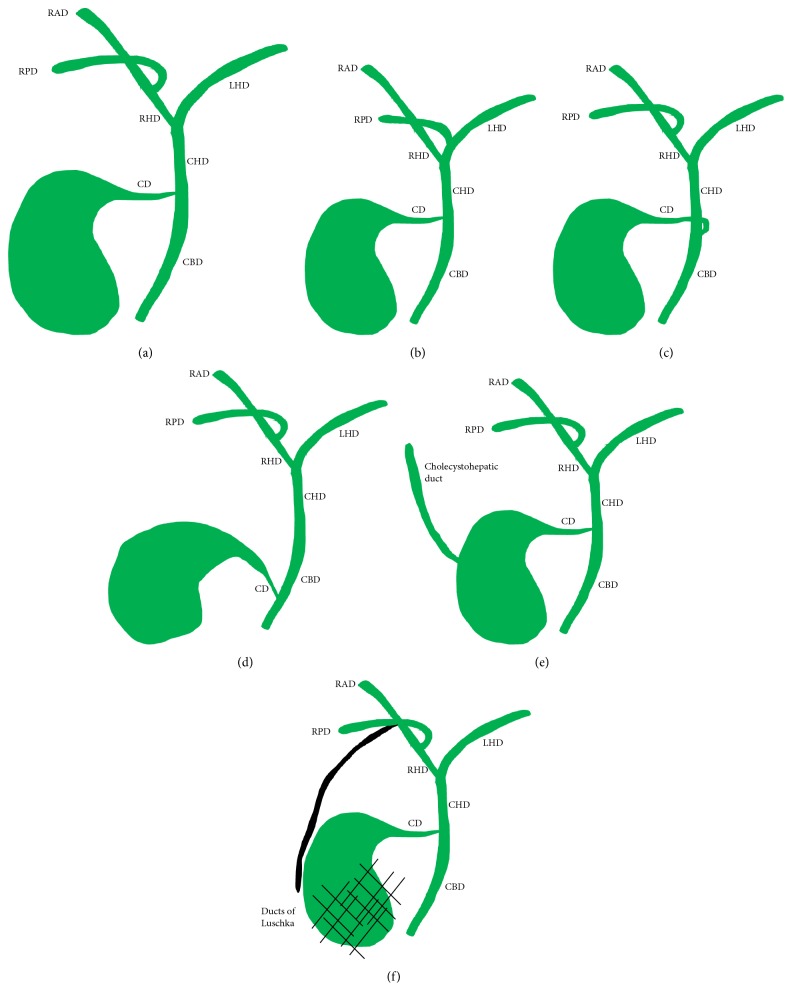
Conventional biliary anatomy and anatomic variants. (a) Conventional biliary anatomy. (b) Aberrant RPD inserting into the left hepatic duct (LHD). (c) Medial insertion of the CD. (d) Low insertion of the CD. (e) Cholecystohepatic duct. (f) Ducts of Luschka (depicted in black).

The intrahepatic ducts course along the portal venous system eventually converging at the portal confluence with the right hepatic duct (RHD) and left hepatic duct (LHD) forming the CHD. The LHD is typically longer than the RHD and drains liver segments II-IV. The RHD is formed by the RPD which drains the posterior segments (VI and VII) and the right anterior duct (RAD) which drains the anterior segments (V and VIII). The RAD and RPD are referred to as sectorial ducts and formed by smaller, more peripheral segmental and subsegmental ducts. On coronal images, the RPD has nearly a horizontal course while the RAD is oriented more vertically [3–5]. The CD should then join the right lateral side of the CHD in the middle third between the confluence and ampulla of Vater to form the CBD [6]. This is considered the conventional anatomy although it is only seen approximately 58-62.6% of the time (Figure 4(a)) [3–5]. Note should also be made of the difference between an aberrant and accessory bile duct. An aberrant duct is one with an anomalous confluence pattern and is the only duct draining a particular segment. An accessory duct is one that is supernumerary and drains a particular segment of the liver in addition to the normal duct [5].

Anomalies of the right biliary ductal system are more common and are typically more relevant for cholecystectomy. The most common right anomaly is an aberrant drainage of the RPD, draining into the LHD, CHD, or CD (in total it represents about 17-18.4%)(Figure 4(b)). If the RPD, RAD, and LHD join at a single location, this is known as a trifurcation anomaly (5-19%). It is also possible for the RHD to drain into the CD or CHD [5, 6].

The CD is extremely variable in its course and insertion. The classic lateral insertion occurs only 51.5-75% of the time (Figure 4(a))[6]. It is important to note medial insertion on the CHD (10-18%), because dissection to its distal end is unsafe, and a longer than normal CD remnant is typically left behind (see Figure 4(c)). Low insertion of the CD (8-11%) may predispose to CD remnant/CBD stone formation which is a potential cause of cholecystectomy syndrome as well as potential problems with CBD stenting (Figure 4(d)). Long parallel course of the cystic duct is defined as close, parallel course to the CHD for at least 2 cm. Often a fibrous sheath will surround the CD and CHD causing their close approximation, which increases risk for extrahepatic duct injury [5, 6]. Sarawagi et al. noted a long parallel course in 7.5% of cases. Other notable but less common cystic duct anomalies include aberrant cystic duct (most commonly inserting into the RHD), double cystic duct, high insertion of the cystic duct, and short cystic duct [6].

Subvesical bile ducts, also referred to as ducts of Luschka, are another clinically important variant. Descriptions of these ducts are not well agreed upon and often contradictory in the literature. Ultimately, subvesical bile ducts are those that pass abnormally close to the gallbladder, within the connective tissue of the GB fossa or that have aberrant/accessory drainage directly into the GB or CD (Figures 4(e) and 4(f)). Cholecystohepatic ducts are also sometimes referred to as cystohepatic or hepaticocholecystic ducts. All subvesical ducts tend to arise from the right ductal system [7, 8]. Cholecystohepatic ducts are rare, occurring in less than 1% of cases, and drain variable amounts of the right hepatic lobe [9]. Regardless of the name subvesical bile ducts are given, description of their location and course is key as with all biliary anomalies.

When evaluating the biliary tree, attention should be given to major intra and extrahepatic ducts and whether the confluence pattern is normal or if there is aberrant insertion. Next determine if there are any accessory ducts draining into the extrahepatic ducts or gallbladder which are not expected. Then follow the course of the CD to its confluence with the extrahepatic duct to ensure it inserts laterally in the middle third. Finally, look closely at the GB fossa to determine if there are any suspicious ducts passing abnormally close to the GB that are at risk of being transected during surgery. These steps will increase the identification of relevant biliary abnormalities and help the surgeon avoid unnecessary postoperative bile leaks.

## 4. Conclusion

This case illustrates a bile duct leak due to a cholecystohepatic duct, a rare biliary anomaly in which an accessory or aberrant bile duct drains directly into the GB lumen or CD. It highlights the importance of recognizing and reporting clinically significant variant biliary duct anatomy. Failure to do so is consequential and may result in a bile duct leak. Thorough knowledge of both normal and variant biliary anatomy as well as an active search pattern by the radiologist should help reduce this risk.
